# EnteroMix and the emerging role of MRNA-based therapeutics in colorectal cancer: from preclinical tumor regression to clinical translation

**DOI:** 10.1097/MS9.0000000000003990

**Published:** 2025-09-29

**Authors:** Muhammad Huzaifa Khalil, Muhammad Daim Hassan, Mahnoor Fatima, Muhammad Talha, Omar Abdullah Gill

**Affiliations:** a Department of Medicine, King Edward Medical University, Lahore, Pakistan; b Shaheed Ziaur Rahman Medical College, Rajshahi Medical University, Bogura, Bangladesh

Colorectal cancer (CRC) remains one of the leading causes of cancer mortality worldwide, with over 1.9 million new cases and 935 000 deaths estimated globally in 2024 alone. Despite advances in surgical interventions, chemotherapy, and targeted therapies, treatment resistance and systemic toxicities limit long-term outcomes. Hence, novel therapeutic modalities, particularly mRNA-based therapeutics, have garnered increasing interest as precision immunotherapies that can potentially improve efficacy and safety profiles^[1]^. This is in line with the TITAN Guidelines on the need for transparency in AI use in healthcare^[2]^.

fmRNA therapeutics harness the body’s own cellular machinery to transiently express therapeutic proteins or antigens that trigger specific immune responses against tumor cells. Recent preclinical and early clinical investigations have demonstrated the promise of this approach in CRC management. Notably, EnteroMix, a personalized mRNA-based cancer vaccine developed using patients’ tumor RNA, exhibited remarkable tumor regression between 60% and 80% in Phase I trials involving 48 CRC patients, alongside 100% immune response activation and an excellent safety profile with no severe adverse events reported. This vaccine represents a departure from conventional chemotherapy and radiotherapy, instead training the immune system to selectively target cancer cells, potentially reducing off-target toxicity^[3-5]^.

The significance of EnteroMix extends beyond CRC. The vaccine platform is also being explored for aggressive cancers such as glioblastoma and melanoma, suggesting broad immunotherapeutic applicability. This aligns with accumulating evidence supporting mRNA technologies for cancer immunotherapy, where mRNA vaccines combined with immune checkpoint inhibitors have shown enhanced tumor regression and prolonged survival in preclinical models. Furthermore, mRNA approaches enable rapid development and personalized treatment adaptations, a critical advantage for heterogeneous malignancies like CRC^[4,5]^.

However, while these early findings are promising, caution remains warranted. Phase I trials primarily assess safety and immunogenicity and lack long-term efficacy data such as progression-free or overall survival. More extensive, randomized trials are urgently needed to validate EnteroMix’s clinical benefits and comparative effectiveness. Additionally, integration of mRNA vaccines into existing therapeutic regimens requires careful optimization to overcome tumor microenvironmental barriers and potential immune evasion^[6-8]^.

In conclusion, EnteroMix, with its transformative potential of mRNA-based therapeutics, represents a new horizon in CRC treatment. The widespread use of the vaccine can be ensured through robust clinical validation and streamlined regulatory approval pathways. Integration of education on mRNA immunotherapy into healthcare policy and cancer care techniques can prepare clinicians for this emerging paradigm. Investment in multidisciplinary research will play a pivotal role in harnessing mRNA vaccines more efficiently, thus increasing the survival rate of CRC patients worldwide.

## Data Availability

Not applicable to this study.

## Research registration unique identifying number (UIN)

No human subjects were involved as the type of research being submitted is an editorial.

## References

[1] Fadlallah H El Masri J Fakhereddine H. Colorectal cancer: recent advances in management and treatment. World J Clin Oncol 2024;15:1136–56. 39351451 10.5306/wjco.v15.i9.1136 PMC11438855

[2] Agha RA Mathew G Rashid R. Transparency in the reporting of Artificial INtelligence – the TITAN guideline. Prem J Sci 2025;10:100082.

[3] Shi Y Shi M Wang Y. Progress and prospects of mRNA-based drugs in pre-clinical and clinical applications. Sig Transduct Target Ther 2024;9:322. 10.1038/s41392-024-02002-z PMC11564800 39543114

[4] Deyhimfar R Izady M Shoghi M. The clinical impact of mRNA therapeutics in the treatment of cancers, infections, genetic disorders, and autoimmune diseases. Heliyon 2024;10:e26971. 38486748 10.1016/j.heliyon.2024.e26971 PMC10937594

[5] Gawalski K Przybyszewska W Hunia J. Unraveling the potential: mRNA therapeutics in oncology. Front Oncol 2025;15:1643444. 40881859 10.3389/fonc.2025.1643444 PMC12381778

[6] Li X Ma S Gao T. The main battlefield of mRNA vaccine - tumor immune microenvironment. Int Immunopharmacol 2022;113:109367. 36327875 10.1016/j.intimp.2022.109367

[7] Li R Hu JC Rong L. The guided fire from within: intratumoral administration of mRNA-based vaccines to mobilize memory immunity and direct immune responses against pathogen to target solid tumors. Cell Discov 2025;10:127. 39743545 10.1038/s41421-024-00743-3 PMC11693766

[8] Gościniak A Eder P Walkowiak J. Artificial gastrointestinal models for nutraceuticals research-achievements and challenges: a practical review. Nutrients 2022;14:2560. 35807741 10.3390/nu14132560 PMC9268564

